# Dr. E. G. Harding

**Published:** 1892-08

**Authors:** 


### Obituary.

Dr. E. G. Harding, of Wyoming, N. Y., was drowned while bathing in the Oatka, on Saturday, July 9, 1892. Dr. Harding was an alumnus of Buffalo University Medical College, a prominent physician in the region of his residence, and will be sadly missed by a large clientèle. In addition to his professional work, he performed the duties of Deacon in the Baptist church, was President of the Board of Education, and prominent in all the business and local interests of his village and vicinity. His funeral was attended on Tuesday, July 12th, by a large concourse of friends, relatives, patrons, and acquaintances. He leaves a wife and a number of children who have the tenderest sympathy of the community.

